# Admission eGFR as a Marker of Systemic Vulnerability in Patients with Spontaneous Intracerebral Hemorrhage: Impact of Premorbid Disability and Acute Kidney Injury on Outcomes

**DOI:** 10.3390/jcm15020562

**Published:** 2026-01-10

**Authors:** Kamil Ludwiniak, Piotr Olejnik, Oliwia Maciejewska, Andrzej Opuchlik, Jolanta Małyszko, Aleksandra Golenia

**Affiliations:** 1 Department of Neurology, University Clinical Center, 02-097 Warsaw, Poland; kamil.ludwiniak@uckwum.pl (K.L.); maciejewska@autograf.pl (O.M.); 2 Department of Neurology, Medical University of Warsaw, 02-091 Warsaw, Poland; piotrek.olejnik2001@gmail.com (P.O.);; 3 Department of Nephrology, Dialysis and Internal Medicine, Medical University of Warsaw, 02-091 Warsaw, Poland; jolmal@poczta.onet.pl

**Keywords:** spontaneous intracerebral hemorrhage, estimated glomerular filtration rate, acute kidney injury, short-term outcomes, mortality

## Abstract

**Background**: Kidney dysfunction is common in intracerebral hemorrhage (ICH), but it is unclear whether reduced estimated glomerular filtration rate (eGFR) on admission is an independent driver of short-term outcomes or a marker of overall vulnerability. **Methods**: In this single-center retrospective study, we analyzed the data of consecutive patients with spontaneous ICH. **Results**: Among 276 patients, 92 (33.3%) presented with eGFR < 60 mL/min/1.73 m^2^ on admission. Only 17/92 (18.5%) had documented pre-existing chronic kidney disease (CKD). Acute kidney injury (AKI) occurred more often in patients with eGFR < 60 mL/min/1.73 m^2^ than in those with eGFR ≥ 60 mL/min/1.73 m^2^ (25.0% vs. 10.3%). In survival models, eGFR ≥ 60 mL/min/1.73 m^2^, predicted higher 90-day survival in the baseline model (OR 3.031, *p* = 0.013) but was attenuated after adjustment for age and premorbid modified Rankin Scale (mRS) and was no longer independent after additional adjustment for laboratory markers. Across all models, the National Institutes of Health Stroke Scale (NIHSS) score, hematoma volume, and history of coronary artery disease remained robust predictors. Higher leukocyte count predicted lower survival, whereas higher hemoglobin predicted higher survival. Among survivors, favorable functional outcome was independently associated with lower NIHSS, younger age, lower premorbid mRS, and absence of documented CKD. Admission eGFR category was not independently associated. **Conclusions**: Reduced admission eGFR primarily reflects baseline frailty and systemic derangement rather than an independent determinant of short-term survival after full adjustment, whereas documented CKD is more informative for disability among survivors. AKI occurs more frequently in patients presenting with reduced eGFR, supporting close renal monitoring in acute ICH.

## 1. Introduction

Intracerebral hemorrhage (ICH) accounts for approximately 10–15% of all strokes and remains one of the most devastating forms of cerebrovascular disease [1]. Despite advances in acute stroke management, ICH is still associated with high morbidity and mortality, particularly in individuals over the age of 40 [2]. The case fatality rate approaches 40% within the first month, and nearly half of the survivors remain dependent on others for activities of daily living [1]. The global burden of ICH is rising, driven by the aging population, an increased prevalence of hypertension, and the expanding use of oral anticoagulants and antithrombotic agents [2].

While the initial hematoma volume, location, and expansion are well-established determinants of early neurological deterioration and outcome, growing evidence indicates that systemic factors including comorbidities and organ dysfunction significantly influence prognosis after ICH [3,4]. Among these, kidney impairment attracted increasing attention as a potentially modifiable predictor of poor outcome, and reduced estimated glomerular filtration rate (eGFR) on admission is a common finding in ICH patients [5]. It may represent either pre-existing chronic kidney disease (CKD) or an early manifestation of acute kidney injury (AKI) secondary to the hemodynamic and inflammatory stress accompanying the hemorrhage.

Impaired kidney function was associated not only with greater hematoma volume but also with an increased risk of hematoma expansion, perihematomal edema, and both short- and long-term mortality [5,6]. The underlying mechanisms are multifactorial, including endothelial dysfunction, impaired autoregulation, inflammation, and oxidative stress, all of which constitute shared pathophysiological pathways between the brain and kidney [7,8]. Although these mechanisms were most extensively described in the context of ischemic stroke, including our previous work demonstrating the contribution of oxidative stress-driven injury to secondary brain damage [9], they are increasingly recognized as relevant also to patients with ICH. This overlap underscores that kidney dysfunction may amplify vulnerability to acute cerebral injury regardless of stroke subtype. In this context, understanding the prognostic value of kidney function on admission is crucial for refining risk stratification, therapeutic decision making, and prognostication in patients with ICH. The early identification of kidney dysfunction could help clinicians anticipate complications, manage blood pressure, and adjust the dosing of nephrotoxic medications.

Therefore, the aim of this study was to examine the prognostic significance of kidney dysfunction in patients with ICH by assessing the impact of reduced admission eGFR, a subsequent development of AKI on short-term survival and 90-day functional outcomes.

## 2. Materials and Methods

In this single-center study, we retrospectively analyzed the data of consecutive patients diagnosed with spontaneous ICH hospitalized at the Neurological Intensive Care Unit of the Department of Neurology, Medical University of Warsaw, Poland, between 1 July 2019 and 30 September 2025. Potentially eligible patients were selected based on the patient admission register. The medical records of all consecutive patients with ICH were retrospectively reviewed. We excluded patients with a previous inpatient and outpatient history of AKI. In addition, patients with ICH secondary to thrombolysis and anticoagulant treatment, arteriovenous malformations, trauma, and those with a terminal illness with the life expectancy < 3 months (advanced cancer, end-stage organ failure) were also excluded from the study.

The presented study does not constitute a medical experiment within the meaning of Article 21 paragraph 1 of the Act of 5 December 1996, on the professions of physician and dentist (Journal of Laws of 2018, item 617) and does not require obtaining an opinion from the Bioethics Committee of the Medical University of Warsaw, referred to in Article 29 paragraph 1 of the aforementioned Act. The need for informed consent was waived due to the retrospective nature of the study. However, all patients provided consent to be contacted three months after hospital discharge for a telephone interview assessing their functional outcome using the modified Rankin Scale (mRS), if such contact was necessary.

### 2.1. Data Source

The data were retrospectively collected from the hospital records of patients admitted with spontaneous ICH, as described previously [10]. The following variables were extracted for each patient: demographic information (age, sex), comorbidities (the presence of hypertension, diabetes mellitus, atrial fibrillation, coronary artery disease, a history of stroke and pre-existing CKD), lifestyle factors (a history of smoking and alcohol abuse), laboratory test results, including the first serum creatinine level and eGFR obtained after emergency department arrival and during hospitalization, clinical assessments (baseline blood pressure, hematoma volume and its location; premorbid functional status assessed with mRS, stroke severity as determined with the National Institutes of Health Stroke Scale (NIHSS)—score on admission and at discharge, range 0–42 with higher scores indicating a greater severity and the baseline Glasgow Coma Scale—GCS), in-hospital AKI defined by KDIGO (the Kidney Disease Improving Global Outcomes) creatinine criteria [11], and outcomes (90-day mRS score). Pre-existing CKD was defined as a documented diagnosis of CKD in the medical record prior to the index ICH admission. Patients with admission eGFR < 60 without documented pre-existing CKD were classified as having no known CKD, acknowledging that reduced eGFR could reflect unrecognized CKD or acute/functional impairment.

### 2.2. Creatinine Levels and eGFR

Serum creatinine levels were measured in blood samples obtained on admission to the emergency department, prior to the initiation of the treatment. All measurements were performed in the hospital’s clinical chemistry laboratory as part of standard diagnostic testing. The reference range for serum creatinine was 0.6–1.2 mg/dL. For each patient, the admission creatinine value was used for subsequent analyses. eGFR was calculated using the CKD-EPI (CKD Epidemiology Collaboration) creatinine equation [12]. eGFR was calculated on admission and, for the primary analysis, categorized as <60 vs. ≥60 mL/min/1.73 m^2^. This threshold was selected a priori because it corresponds to CKD stage ≥3 and is widely used as a clinically meaningful cut-off associated with adverse outcomes. In addition, dichotomizing the variables enhanced result interpretability and maintained sufficient event numbers within each category for multivariable analysis. To reduce loss of information, eGFR was also summarized as a continuous measure in baseline characteristics.

AKI was assessed by monitoring serum creatinine levels repeatedly during hospitalization as part of routine care in the Neurological Intensive Care Unit. AKI was defined according to the KDIGO creatinine criteria as (1) an increase in serum creatinine by ≥0.3 mg/dL within 48 h, or (2) an increase to ≥1.5 times baseline within 7 days. Baseline creatinine was defined as the first creatinine obtained on admission [11]. Urine output criteria were not systematically applied due to incomplete data.

In-hospital complications and treatment exposures were not included as predictors in multivariable outcome models because of their time-dependent nature and the potential for indication bias and survivorship bias in standard logistic regression analyses.

### 2.3. Imaging Analysis

Computed tomography was used to identify patients with ICH, and scans were evaluated by neuroradiologists as part of standard care. Additionally, ICH hematoma volume was measured on the initial non-contrast brain CT scan by a study investigator blinded to clinical data and creatinine status, using the ABC/2 method, with *A* representing the greatest diameter on the largest hemorrhage slice, *B* being the diameter perpendicular to *A*, and *C* being the approximate number of axial slices containing hemorrhage multiplied by the slice thickness [13]. ICH was classified as lobar in location if it stemmed from the cerebral hemispheres, superficial to the deep gray matter structures. Hemorrhage originating from the thalamus or basal ganglia were classified as deep, whereas those arising in the brainstem or cerebellum were classified as infratentorial.

### 2.4. Clinical Outcomes

Clinical outcomes included the 90-day functional status assessed using the modified mRS based on medical records or structured telephone interviews. For the purposes of regression analysis, the primary endpoint was survival at 90 days, defined as an mRS score of 0–5, whereas death was defined as the mRS score of 6 [4]. Secondary outcome analyses were performed among 90-day survivors to examine disability beyond mortality, dichotomizing functional status as favorable (mRS 0–3) versus poor (mRS 4–5).

### 2.5. Statistical Analysis

Statistical analyses were performed using IBM SPSS Statistics, version 29. The Shapiro–Wilk test was used to assess the normality of continuous variables. Continuous variables were presented as the median (interquartile range) and compared between groups using the Mann–Whitney U test. Categorical variables were presented as counts (percentages) and compared using the χ^2^ test of independence. Multivariable logistic regression was used to identify independent predictors of 90-day outcomes. Six sequential models were fitted to assess the robustness of the association between admission kidney function and clinical outcomes: Models A–C evaluated 90-day survival (mRS 0–5 vs. 6), and Models D–F evaluated functional outcome among 90-day survivors (favorable mRS 0–3 vs. poor mRS 4–5), using the same stepwise adjustment strategy and covariate sets as in Models A–C. Model A included admission clinical severity and hemorrhage burden (NIHSS, GCS, hematoma volume), admission eGFR category (<60 vs. ≥60 mL/min/1.73 m^2^), vascular comorbidities, and admission blood pressure. Model B additionally adjusted for age and premorbid mRS. Model C further included admission laboratory variables. Results are reported as odds ratios (ORs) with 95% confidence intervals (CIs). Admission eGFR was specified a priori as a dichotomous variable (<60 vs. ≥60 mL/min/1.73 m^2^). A sensitivity analysis modeling eGFR as a continuous predictor was performed and is reported in the Supplementary Materials. All tests were two-sided and *p* < 0.05 was considered statistically significant.

## 3. Results

### 3.1. General Characteristics

Among 276 patients with ICH, 92 (33.3%) presented with decreased eGFR (<60 mL/min/1.73 m^2^). Those patients were older (the mean age 74.12 years) and carried a substantially greater comorbidity burden. Diabetes (28.3% vs. 15.8%), prior stroke (27.2% vs. 9.8%), and CKD (18.5% vs. 2.2%) were all significantly more common in the reduced eGFR group. Their premorbid disability was more severe (the median mRS 2 vs. 1). Notably, among the 92 patients with eGFR < 60 mL/min/1.73 m^2^, only 17 (18.5%) had documented pre-existing CKD, whereas 75 (81.5%) had no known CKD at baseline. Because pre-admission creatinine and eGFR values were not consistently available, we could not reliably distinguish previously unrecognized CKD from acute kidney dysfunction at presentation. Therefore, we report documented CKD history separately and interpret admission eGFR primarily as a prognostic biomarker at presentation. The demographic and clinical characteristics of the study group are summarized in Table 1.

### 3.2. Admission eGFR and Baseline Variables

Laboratory abnormalities reflected impaired kidney reserve, including higher admission glucose (185.60 ± 123 mg/dL vs. 151.67 ± 60.47 mg/dL; *p* = 0.01), urea (56.80 ± 30.28 mg/dL vs. 34.35 ± 12.08 mg/dL; *p* = 0.001), and potassium (4.24 ± 0.72 mmol/L vs. 3.91 ± 0.49 mmol/L; *p* = 0.001), alongside lower hemoglobin (13.4 ± 2.23 g/dL vs. 13.98 ± 1.82 g/dL; *p* = 0.005) and hematocrit (39.58 ± 6.17% vs. 40.84 ± 5.05%; *p* = 0.32). They also presented with more severe neurological deficits, exhibiting lower GCS (13 vs. 14; *p* = 0.012) and higher NIHSS scores (16.99 ± 12.76 vs. 13.52 ± 12.17; *p* = 0.029) on admission. Hematoma volume and location were comparable between the groups (*p* = 0.52); (Table 1).

### 3.3. In-Hospital AKI and Discharge Creatinine

Patients with decreased admission eGFR developed AKI more often (25.0% vs. 10.3%, *p* = 0.001), and discharge creatinine levels remained higher (1.67 ± 1.55 mg/dL vs. 0.92 ± 0.58 mg/dL; *p* = 0.001).

### 3.4. Logistic Regression Model

In Model A, a higher NIHSS score (OR 0.848, 95% CI 0.79–0.91; *p* < 0.001), larger admission hematoma volume (OR 0.979, 95% CI 0.96–1.00; *p* = 0.032), and a history of coronary artery disease (OR 0.134, 95% CI 0.03–0.52; *p* = 0.004) were independently associated with lower odds of survival. Admission eGFR ≥ 60 mL/min/1.73 m^2^was associated with higher odds of survival compared with eGFR < 60 mL/min/1.73 m^2^ (OR 3.031, 95% CI 1.26–7.29; *p* = 0.013) (Table 2).

In Model B, the NIHSS score (OR 0.838, 95% CI 0.77–0.91; *p* < 0.001), hematoma volume (OR 0.973, 95% CI 0.95–0.99; *p* = 0.012), and a history of coronary artery disease (OR 0.119, 95% CI 0.03–0.54; *p* = 0.006) remained robust predictors, while premorbid mRS emerged as an independent predictor of reduced survival (OR 0.587, 95% CI 0.40–0.87; *p* = 0.008). Age was not statistically significant (OR 0.969, 95% CI 0.94–1.01; *p* = 0.097).

After adjustment for age and premorbid mRS, the association between eGFR ≥ 60 mL/min/1.73 m^2^ and survival was attenuated and no longer statistically significant (OR 2.070, 95% CI 0.80–5.38; *p* = 0.136). A documented history of CKD showed wide CIs and was borderline after adjustment (*p* = 0.056), indicating an imprecise estimate (Table 2).

In Model C, which additionally included admission laboratory parameters (glucose, urea, sodium, potassium, leukocyte count, platelet count, hemoglobin, hematocrit, and C-reactive protein), a higher NIHSS score on admission (OR 0.815, 95% CI 0.74–0.90; *p* < 0.001), larger hematoma volume (OR 0.974, 95% CI 0.95–1.00; *p* = 0.025), and a history of coronary artery disease (OR 0.080, 95% CI 0.01–0.45; *p* = 0.004) remained independently associated with lower odds of survival.

Among the laboratory measures, a higher leukocyte count was associated with lower survival (OR 0.816, 95% CI 0.70–0.95; *p* = 0.007), whereas higher hemoglobin levels were associated with higher survival (OR 2.059, 95% CI 1.06–4.00; *p* = 0.033). Age was independently associated with lower survival (OR 0.949, 95% CI 0.91–0.99; *p* = 0.019), while premorbid mRS showed a non-significant trend (*p* = 0.082). In this extended laboratory model, categorical admission eGFR (≥60 vs. <60 mL/min/1.73 m^2^) was not independently associated with survival (OR 1.467, 95% CI 0.44–4.90; *p* = 0.533), and other laboratory parameters did not show independent associations after full adjustment (Table 2).

Among 90-day survivors, in multivariable logistic regression without adjustment for age and premorbid disability (Model D), a higher NIHSS score on admission was associated with lower odds of a favorable functional outcome (OR 0.866; 95% CI 0.79–0.95; *p* = 0.003). Hypertension (OR 0.285; 95% CI 0.08–0.97; *p* = 0.045), atrial fibrillation (OR 0.141; 95% CI 0.03–0.59; *p* = 0.007), and documented CKD (OR 0.209; 95% CI 0.05–0.92; *p* = 0.038) were also associated with reduced odds of achieving mRS 0–3 (Table 3).

After additional adjustment for age and premorbid mRS (Model E), a higher NIHSS score remained independently associated with lower odds of a favorable functional outcome (OR 0.839; 95% CI 0.75–0.94; *p* = 0.003), while older age (OR 0.960; 95% CI 0.92–1.00; *p* = 0.037), higher premorbid mRS (OR 0.574; 95% CI 0.36–0.91; *p* = 0.019), and documented CKD (OR 0.177; 95% CI 0.04–0.87; *p* = 0.033) were independent predictors of lower odds of favorable functional outcome. Admission eGFR category was not independently associated with outcome (Table 3).

In an extended model additionally adjusting for admission laboratory parameters, both NIHSS (OR 0.831; 95% CI 0.72–0.96; *p* = 0.012) and CKD (OR 0.143; 95% CI 0.02–0.86; *p* = 0.034) remained significant predictors, while higher admission glucose levels were associated with lower odds of a favorable functional outcome (OR 0.977; 95% CI 0.96–0.99; *p* = 0.001). Across all model specifications, the admission eGFR category was not independently associated with functional outcome (Table 3).

In Model F, diabetes mellitus was associated with markedly higher odds of a favorable outcome (OR 16.43, 95% CI 2.18–123.62; *p* = 0.007). However, the very wide confidence interval and the fact that this effect appeared only after inclusion of laboratory covariates suggest an unstable estimate. Therefore, this finding should be interpreted cautiously and not regarded as evidence of a protective effect (Table 3).

As a sensitivity analysis, admission eGFR was additionally modeled as a continuous predictor. The direction and significance of associations were consistent with the primary dichotomous eGFR analysis (<60 vs. ≥60 mL/min/1.73 m^2^). Full model outputs are provided in Supplementary Tables S1 and S2.

## 4. Discussion

In this retrospective, single-center cohort study, we assessed whether kidney vulnerability at presentation and kidney-related events during hospitalization were associated with short-term outcomes after ICH. Patients with reduced eGFR on admission were older, had a higher comorbidity burden and greater premorbid disability, presented with more severe neurological deficits and more pronounced biochemical disturbances. Impaired kidney function on admission was a heterogeneous condition: only a minority of patients with eGFR < 60 mL/min/1.73 m^2^ had documented CKD, which limits causal inference and supports the view that reduced eGFR is, at least in part, a marker of global frailty, vascular burden, and acute systemic derangement rather than a kidney-specific independent causal determinant of mortality.

In multivariable survival analyses, higher admission eGFR (≥60 mL/min/1.73 m^2^) initially was associated with better 90-day survival, but this association attenuated and became non-significant after additional adjustment for age and premorbid mRS, while premorbid disability remained independently prognostic. After adding admission laboratory variables, the prognostic signal shifted toward markers of systemic vulnerability: leukocytosis was associated with lower survival and higher hemoglobin with better survival, further reducing the independent contribution of eGFR. Among survivors, functional outcome (mRS 0–3 at 90 days) was primarily determined by NIHSS score, age, and premorbid mRS. Importantly, documented CKD, rather than the admission eGFR category, remained independently associated with worse functional recovery, consistent with CKD reflecting longer-term systemic or vascular burden.

The observations are consistent with prior studies showing that kidney impairment is common in individuals with ICH and contributes to clinical instability and early deterioration. Molshatzki et al. showed that moderate to severe kidney impairment in acute ICH was associated with larger hematoma volume and nearly six-fold higher 1-year mortality compared to patients without CKD [5]. Similarly, Zheng et al. reported that decreased eGFR independently predicted poor outcome in acute ICH, regardless of blood pressure management strategy [14]. Additionally, large observational cohorts and meta-analyses have confirmed that CKD was associated with higher mortality and poorer functional outcomes after ICH, even after adjustment for age and vascular risk factors [15]. Beyond dichotomous thresholds, several authors examined eGFR as a continuous prognostic marker. In a population-based analysis, lower eGFR was associated with higher 1-month and 1-year mortality after adjustment for sex, age, stroke severity, and comorbidities [16]. In our cohort, lower eGFR together with higher NIHSS scores and poorer premorbid mRS suggests that admission kidney function captures vascular vulnerability and overall physiological reserve (e.g., atherosclerosis, endothelial dysfunction and impaired autoregulation) at the time of ICH. Moreover, we observed no significant differences in baseline hematoma volume or location across eGFR categories, suggesting that, the association between reduced eGFR and outcome was driven primarily by frailty, comorbidity burden, and acute physiological stress rather than by hemorrhage characteristics.

Kidney injury during hospitalization proved to be even more prognostically important. In line with our earlier research demonstrating that AKI developed in 13.5% of ICH patients and markedly worsened short-term survival [10], we found that in-hospital AKI was among the strongest independent predictors of 90-day mortality. The finding is also supported by other reports, including a recent mini-review summarizing higher mortality and neurological decline in patients who developed AKI after ICH [17], as well as a study by Burgess et al., which showed that AKI occurred more frequently in patients undergoing intensive blood pressure reduction, especially those with underlying CKD [18]. Together, these data highlight AKI as a key component of systemic destabilization in ICH and a potential therapeutic target.

These findings fit the concept of shared kidney–brain vulnerability, in which microvascular injury and inflammatory stress, encompassing endothelial dysfunction, impaired autoregulation, inflammation, and oxidative stress, may contribute to early deterioration [17]. The associations between metabolic and inflammatory markers (urea, glucose, leukocytosis), neurological severity, and mortality further underscore the importance of the systemic response in shaping outcomes after ICH [19]. Taken together, our data support approaching ICH as an acute brain injury with important multisystem consequences, where early recognition of kidney vulnerability and close monitoring for AKI may help optimize management.

This study has several limitations that should be acknowledged. First, baseline kidney function prior to admission was not available for all participants; thus, in patients without known CKD, reduced admission eGFR may reflect either acute kidney dysfunction or previously unrecognized CKD. Second, although we used a clinically established threshold (<60 mL/min/1.73 m^2^) for the primary analysis, dichotomization may attenuate dose–response relationships. Therefore, results should be interpreted in that context. Third, the retrospective, single-center design carries the typical risk of selection bias and limits the generalizability of the results. Patterns of referral, local treatment protocols, and thresholds for diagnostic neuroimaging may differ across institutions, which could influence both kidney outcomes and outcome assessments. Next, although we captured a wide range of clinical and laboratory variables, unmeasured confounders such as dehydration status, chronic medication use (e.g., angiotensin-converting enzyme inhibitors, diuretics), or undiagnosed CKD may have contributed to both decreased eGFR on admission and the likelihood of developing AKI. Moreover, our assessment of kidney function relied on serum creatinine-based eGFR equations, which are imperfect in the context of acute illness, sarcopenia, or hemodynamic instability [20]. Serum creatinine may underestimate kidney dysfunction in frail or cachectic patients and may fluctuate with acute systemic stress, potentially misclassifying some individuals. We did not evaluate albuminuria or other markers of chronic kidney damage, which may provide a more nuanced picture of baseline kidney health and prognostic value. Furthermore, although AKI was defined using the KDIGO criteria [11], urine output data were incomplete, meaning some AKI episodes may have gone undetected. Additionally, our cohort size, while adequate for the primary analyses, limits the statistical power to explore interactions and less frequent events (e.g., AKI stages or subgroup effects). However, these limitations do not diminish the overall message but highlight that prospective, multicenter studies with standardized kidney and neurological assessments are needed to better define how kidney dysfunction shapes the trajectory of ICH.

## 5. Conclusions

In this ICH cohort, reduced admission eGFR mainly marked overall vulnerability rather than an independent determinant of short-term survival after full adjustment, whereas documented CKD was linked to worse functional recovery among survivors. These findings support early identification of renal vulnerability and close monitoring for in-hospital AKI. Interpretation should be cautious because pre-admission kidney function was unavailable for many patients and because dichotomizing eGFR at <60 mL/min/1.73 m^2^ may have obscured dose–response effects.

## Figures and Tables

**Table 1:** Baseline characteristics of patients with ICH stratified by admission eGFR.

Variables	Total (n = 276)	Normal Admission eGFR (≥60) * (n = 184)	Decreased Admission eGFR (<60) * (n = 92)	*p*-Value *
Demographics				
Age (years), mean ± SD [Q1; Q3]	68.95 ± 16.52 [55.00; 83.00]	66.36 ± 16.06 [53.00; 80.75]	74.12 ± 16.28 [61.50; 86.75]	0.001 ^a,^**
Male sex, n, (%)	137 (49.6)	90 (48.9)	47 (51.1)	0.733 ^b^
Comorbidities				
Hypertension, n, (%)	206 (74.6)	133 (72.3)	73 (79.3)	0.203 ^b^
Diabetes mellitus, n, (%)	55 (19.9)	29 (15.8)	26 (28.3)	0.014 ^b,^**
Atrial fibrillation, n, (%)	24 (8.7)	13 (7.1)	11 (12.0)	0.174 ^b^
Coronary artery disease, n, (%)	32 (11.6)	19 (10.3)	13 (14.1)	0.351 ^b^
History of stroke, n, (%)	43 (15.6)	18 (9.8)	25 (27.2)	0.001 ^b,^**
Chronic kidney disease, n, (%)	21 (7.6)	4 (2.2)	17 (18.5)	0.001 ^b,^**
Smoking, n, (%)	52 (18.8)	37 (20.1)	15 (16.3)	0.446 ^b^
Alcohol abuse, n, (%)	34 (12.3)	19 (10.3)	15 (16.3)	0.154 ^b^
Prior antiplatelet use, n, (%)	55 (19.9)	32 (17.4)	23 (25.0)	0.136 ^b^
Premorbid mRS score, median [Q1; Q3]	1 [0.00; 3.00]	1 [0.00; 2.00]	2 [1.00; 3.00]	0.001 ^a,^**
Admission variables				
Glucose (mg/dL) mean ± SD [Q1; Q3]	162.95 ± 87.72 [113.00; 188.00]	151.67 ± 60.47 [110.00; 180.25]	185.60 ± 123 [120.00; 210.25]	0.010 ^a,^**
Urea (mg/dL) mean ± SD [Q1; Q3]	41.84 ± 22.65 [30.00; 46.00]	34.35 ± 12.08 [26.00; 40.00]	56.80 ± 30.28 [40.00; 64.75]	0.001 ^a,^**
Potassium (mmol/L) mean ± SD [Q1; Q3]	4.02 ± 0.59 [3.69; 4.31]	3.91 ± 0.49 [3.65; 4.22]	4.24 ± 0.72 [3.81; 4.62]	0.001 ^a,^**
Sodium (mmol/L) mean ± SD [Q1; Q3]	139.52 ± 7.78 [138.00; 141.90]	139.35 ± 9 [138.03; 141.78]	139.86 ± 4.44 [137.70; 142.28]	0.432 ^a^
Leukocytes (×10^3^/µL) mean ± SD [Q1; Q3]	10.54 ± 4.80 [7.60; 12.00]	10.28 ± 4.85 [7.65; 11.57]	11.06 ± 4.70 [7.58; 13.49]	0.162 ^a^
Platelet count (×10^3^/µL) mean ± SD [Q1; Q3]	226.09 ± 77.68 [173.00; 262.75]	220.68 ± 73.33 [175.50; 260.75]	236.92 ± 85.11 [171.25; 278.50]	0.173 ^a^
Hemoglobin (g/dL) mean ± SD [Q1; Q3]	13.79 ± 1.98 [12.80; 15.00]	13.98 ± 1.82 [13.20; 15.10]	13.40 ± 2.23 [12.23; 15.00]	0.005 ^a,^**
Hematocrit (%) mean ± SD [Q1; Q3]	40.42 ± 5.47 [37.30; 43.58]	40.84 ± 5.05 [38.00; 43.30]	39.58 ± 6.17 [36.00; 44.00]	0.032 ^a,^**
C-reactive protein (mg/L) mean ± SD [Q1; Q3]	12.44 ± 30.32 [1.10; 10.33]	10.11 ± 24.26 [1.00; 7.48]	17.10 ± 39.59 [1.30; 14.93]	0.174 ^a^
Creatinine on admission (mg/dL) mean ± SD [Q1; Q3]	1.12 ± 0.81 [0.79; 1.17]	0.88 ± 0.20 [0.73; 1.02]	1.66 ± 1.23 [1.04; 1.57]	0.001 ^a,^**
eGFR (mL/min/1.73 m^3^) mean ± SD [Q1; Q3]	68.93 ± 22.38 [54.00; 84.75]	81.39 ± 14.3 [69.00; 93.00]	44.02 ± 13.05 [39.00; 54.00]	N/A ***
Glasgow Coma Scale on admission, median [Q1; Q3]	14 [8.00; 15.00]	14 [9.00; 15.00]	13 [6.00; 15.00]	0.012 ^a,^**
NIHSS score on admission (points)	14.67 ± 12.45 [3.00; 22.00]	13.52 ± 12.17 [3.00; 20.00]	16.99 ± 12.76 [5.00; 25.00]	0.029 ^a,^**
Admission hematoma volume (mL)	28.49 ± 39.10 [3.66; 41.63]	26.72 ± 38.21 [3.97; 34.07]	32.02 ± 40.81 [3.40; 50.55]	0.520 ^a^
Hematoma location (n, %)				0.925 ^b^
lobar,	96 (34.8)	64 (34.8)	32 (34.8)	
deep,	144 (52.2)	95 (51.6)	49 (53.3)	
infratentorial	36 (13.0)	25 (13.6)	11 (12.0)	
Intraventricular hemorrhage, n (%)	103 (37.3)	63 (34.2)	40 (43.5)	0.135 ^b^
Baseline blood pressure, mean ± SD [Q1; Q3]				
Systolic blood pressure (mmHg) mean ± SD [Q1; Q3]	167.83 ± 35.96 [143.00; 188.00]	166.36 ± 34.57 [142.00; 183.75]	170.75 ± 38.64 [143.25; 191.50]	0.343 ^a^
Diastolic blood pressure (mmHg) mean ± SD [Q1; Q3]	94.51 ± 22.88 [79.00; 107.00]	95.09 ± 21.93 [80.00; 106.00]	93.36 ± 24.75 [75.25 ± 110.00]	0.528 ^a^

n: number of patients; SD: standard deviation; Q1: quartile 25′; Q3: quartile 75′; eGFR—estimated glomerular filtration rate; NIHSS—National Institutes of Health Stroke Scale; mRS—modified Rankin Scale; ^a^ U Mann–Whitney test; ^b^ Chi-Squared Χ^2^; * compared sub-populations; ** *p* < 0.05; *** for admission eGFR, *p*-values were not calculated because subgroups were defined by the eGFR threshold (<60 vs. ≥60 mL/min/1.73 m^2^).

**Table 2:** Logistic regression analysis of survival in patients with ICH.

	Survival Group (mRS 0–5)								
	Model A			Model B			Model C		
	OR	95% CI	*p*	OR	95% CI	*p*	OR	95% CI	*p*
Clinical variables									
NIHSS score on admission	0.848	0.79–0.91	0.001 *	0.838	0.77–0.91	0.001 *	0.815	0.74–0.90	0.001 *
Admission hematoma volume (mL)	0.979	0.96–1.00	0.032 *	0.973	0.95–0.99	0.012 *	0.974	0.95–1.00	0.025 *
Admission eGFR ≥ 60 vs. <60 mL/min/1.73 m^2^	3.031	1.26–7.29	0.013 *	2.070	0.80–5.38	0.136	1.467	0.44–4.90	0.533
Glasgow Coma Scale on admission	1.183	1.00–1.40	0.054	1.165	0.97–1.40	0.095	1.087	0.89–1.33	0.412
Hypertension	1.895	0.74–4.88	0.186	2.498	0.88–7.10	0.086	2.570	0.79–8.41	0.119
Diabetes mellitus	0.479	0.17–1.32	0.153	0.381	0.12–1.17	0.092	0.702	0.16–3.01	0.634
Atrial fibrillation	1.195	0.30–4.80	0.802	2.503	0.53–11.91	0.249	2.821	0.45–17.78	0.270
Coronary artery disease	0.134	0.03–0.52	0.004 *	0.119	0.03–0.54	0.006 *	0.080	0.01–0.45	0.004 *
History of stroke	0.666	0.21–2.12	0.491	1.072	0.28–4.10	0.919	1.005	0.19–5.21	0.995
Chronic kidney disease	8.320	1.19–58.35	0.033 *	8.933	0.95–84.20	0.056	8.875	0.75–105.06	0.083
Smoking	1.489	0.50–4.45	0.476	1.187	0.38–3.72	0.768	1.292	0.34–4.92	0.707
Alcohol abuse	0.378	0.09–1.55	0.176	0.75	0.06–1.20	0.085	0.282	0.04–1.80	0.181
Systolic blood pressure on admission (mmHg)	1.002	0.98–1.02	0.839	1.007	0.99–1.03	0.498	1.008	0.99–1.03	0.482
Diastolic blood pressure on admission (mmHg)	1.008	0.98–1.04	0.575	0.993	0.96–1.02	0.632	0.983	0.95–1.02	0.319
Age (years)	--			0.969	0.94–1.01	0.097	0.949	0.91–0.99	0.019 *
Premorbid mRS	--			0.587	0.40–0.87	0.008 *	0.672	0.43–1.05	0.082
Admission laboratory variables (Model C only)									
Glucose (mg/dL)	--			--			0.992	0.98–1.00	0.116
Urea (mg/dL)	--			--			0.984	0.96–1.01	0.206
Potassium (mmol/L)	--			--			1.234	0.49–3.11	0.656
Sodium (mmol/L)	--			--			0.994	0.91–1.09	0.905
Leukocyte count (×10^3^/uL)	--			--			0.816	0.70–0.95	0.007 *
Platelet count (×10^3^/uL)	--			--			1.006	1.00–1.01	0.145
Hemoglobin (g/dL)	--			--			2.059	1.06–4.00	0.033 *
Hematocrit (%)	--			--			0.849	0.68–1.07	0.161
C-reactive protein (mg/L)	--			--			1.015	1.00–1.03	0.081

OR—odds ratio; CI—confidence interval; ICH—intracerebral hemorrhage; eGFR—estimated glomerular filtration rate; NIHSS—National Institutes of Health Stroke Scale; mRS—modified Rankin Scale; * *p* < 0.05; -- not included in the model.

**Table 3:** Logistic regression analysis of favorable outcome in patients with ICH.

	Favorable Outcome (mRS 0–3)								
	Model D			Model E			Model F		
	OR	95% CI	*p*	OR	95% CI	*p*	OR	95% CI	*p*
Clinical variables									
NIHSS score on admission	0.866	0.79–0.95	0.003 *	0.839	0.75–0.94	0.003 *	0.831	0.72–0.96	0.012 *
Admission hematoma volume (mL)	0.978	0.95–1.01	0.140	0.969	0.94–1.00	0.051	0.964	0.93–1.00	0.068
Admission eGFR ≥ 60 vs. <60 mL/min/1.73 m^2^	0.732	0.26–2.08	0.558	0.521	0.18–1.55	0.241	0.661	0.18–2.50	0.541
Glasgow Coma Scale on admission	0.974	0.73–1.30	0.861	0.899	0.66–1.23	0.507	0.981	0.67–1.44	0.921
Hypertension	0.285	0.08–0.97	0.045 *	0.392	0.11–1.38	0.144	0.395	0.08–1.94	0.252
Diabetes mellitus	2.094	0.62–7.13	0.237	2.328	0.60–8.97	0.220	16.429	2.18–123.62	0.007 *
Atrial fibrillation	0.142	0.03–0.59	0.007 *	0.285	0.06–1.41	0.124	0.202	0.02–1.68	0.139
Coronary artery disease	1.701	0.31–9.27	0.539	1.357	0.24–7.64	0.729	1.643	0.19–14.34	0.653
History of stroke	0.972	0.30–3.16	0.963	1.921	0.49–7.59	0.352	0.784	0.15–3.98	0.769
Chronic kidney disease	0.209	0.05–0.92	0.038 *	0.177	0.04–0.87	0.033 *	0.143	0.02–0.86	0.034 *
Smoking	0.592	0.20–1.75	0.342	0.476	0.15–1.53	0.212	0.206	0.05–0.88	0.034 *
Alcohol abuse	1.449	0.31–6.80	0.638	0.989	0.19–5.29	0.989	1.912	0.23–16.23	0.552
Systolic blood pressure on admission (mmHg)	1.007	0.98–1.03	0.567	1.016	0.99–1.04	0.255	1.036	1.00–1.07	0.380
Diastolic blood pressure on admission (mmHg)	1.008	0.92–1.05	0.670	0.982	0.94–1.02	0.374	0.971	0.93–1.02	0.232
Age (years)	--			0.960	0.92–1.00	0.037 *	0.951	0.90–1.00	0.065
Premorbid mRS	--			0.574	0.36–0.91	0.019 *	0.558	0.31–1.00	0.050
Admission laboratory variables (Model F only)									
Glucose (mg/dL)	--			--			0.977	0.96–0.99	0.001 *
Urea (mg/dL)	--			--			0.986	0.94–1.04	0.578
Potassium (mmol/L)	--			--			3.323	1.00–11.00	0.050
Sodium (mmol/L)	--			--			1.119	0.95–1.32	0.174
Leukocyte count (×10^3^/uL)	--			--			0.998	0.81–1.23	0.987
Platelet count (×10^3^/uL)	--			--			0.999	0.99–1.01	0.777
Hemoglobin (g/dL)	--			--			1.102	0.39–3.15	0.856
Hematocrit (%)	--			--			1.106	0.74–1.66	0.628
C-reactive protein (mg/L)	--			--			1.000	0.98–1.02	0.995

OR—odds ratio; CI—confidence interval; eGFR—estimated glomerular filtration rate; NIHSS—National Institutes of Health Stroke Scale; mRS—modified Rankin Scale; * *p* < 0.05; -- not included in the model.

## Data Availability

The datasets used and analyzed during the current study are available from the corresponding author on reasonable request.

## Supplementary Materials

The following supporting information can be downloaded at https://www.mdpi.com/article/10.3390/jcm15020562/s1, Table S1. Logistic regression analysis of survival in patients with ICH; Table S2. Logistic regression analysis of favorable outcome in patients with ICH.
