# Supplementary material for: Admission eGFR as a Marker of Systemic Vulnerability in Patients with Spontaneous Intracerebral Hemorrhage: Impact of Premorbid Disability and Acute Kidney Injury on Outcomes
Source: J Clin Med. 2026 Jan 10;15(2):562. doi: 10.3390/jcm15020562 (PMC12842251; doi:10.3390/jcm15020562)
Supplement: Supplementary file 1 [file jcm-15-00562-s001.zip › jcm-4080602-supplementary.pdf]

Table S1. Logistic regression analysis of survival in patients with ICH

	Survival group (mRS 0-5)								
	Model 1			Model 2			Model 3		
	OR	95% CI	*p*	OR	95% CI	*p*	OR	95% CI	*P*
**Clinical variables**									
NIHSS score on admission	0.850	0.79-0.92	0.001*	0.840	0.78-0.91	0.001*	0.815	0.74-0.90	0.001*
Admission hematoma volume (ml)	0.980	0.96-1.00	0.040*	0.973	0.95-0.99	0.011*	0.974	0.95-1.00	0.020*
Admission eGFR (per 10 mL/min/1.73 m²)	1.025	1.00-1.05	0.017*	1.012	0.99-1.04	0.277	0.990	0.96-1.02	0.550
Glasgow Coma Scale on admission	1.191	1.00-1.41	0.044*	1.169	0.98-1.40	0.088	1.087	0.89-1.33	0.408
Hypertension	1.931	0.75-4.98	0.173	2.532	0.89-7.22	0.082	2.417	0.73-7.98	0.148
Diabetes mellitus	0.438	0.16-1.21	0.110	0.360	0.12-1.10	0.074	0.756	0.18-3.24	0.707
Atrial fibrillation	1.352	0.33-5.59	0.677	2.607	0.55-12.38	0.228	2.749	0.44-17.25	0.280
Coronary artery disease	0.170	0.05-0.64	0.009*	0.139	0.03-0.60	0.008*	0.087	0.02-0.47	0.005*
History of stroke	0.650	0.20-2.10	0.472	1.062	0.28-4.07	0.929	0.948	0.18-4.99	0.950
Chronic kidney disease	8.569	1.18-62.47	0.034*	8.134	0.87-76.23	0.066	6.185	0.54-71.25	0.144
Smoking	1.358	0.45-4.14	0.591	1.152	0.37-3.63	0.809	1.384	0.36-5.26	0.634
Alcohol abuse	0.399	0.10-1.67	0.208	0.282	0.07-1.22	0.091	0.316	0.05-2.01	0.223
Systolic blood pressure on admission (mmHg)	1.003	0.98-1.02	0.772	1.006	0.99-1.03	0.515	1.006	0.99-1.03	0.575
Diastolic blood pressure on admission (mmHg)	1.008	0.98-1.04	0.577	0.993	0.96-1.02	0.658	0.985	0.95-1.02	0.363
Age (years)	--			0.970	0.94-1.01	0.109	0.944	0.90-0.99	0.013*
Premorbid mRS	--			0.581	0.39-0.86	0.007*	0.675	0.43-1.06	0.085
**Admission laboratory variables (Model C only)**									
Glucose (mg/dL)	--			--			0.992	0.98-1.00	0.112
Urea (mg/dL)	--			--			0.976	0.95-1.01	0.105
Potassium (mmol/L)	--			--			1.110	0.43-2.84	0.828
Sodium (mmol/L)	--			--			0.987	0.89-1.10	0.811
Leukocyte count (x10^3/uL)	--			--			0.819	0.71-0.95	0.008*
Platelet count (x10^3/uL)	--			--			1.005	1.00-1.01	0.181
Hemoglobin (g/dL)	--			--			2.184	1.10-4.36	0.027*
Hematocrit (%)	--			--			0.843	0.67-1.06	0.151
C-reactive protein (mg/L)	--			--			1.016	1.00-1.03	0.061

OR - odds ratio; CI - confidence interval; ICH - intracerebral hemorrhage; eGFR- estimated glomerular filtration rate; NIHSS - National Institutes of Health Stroke Scale; mRS – modified Rankin Scale; *p < 0.05; -- not included in the model.

Table S2. Logistic regression analysis of favorable outcome in patients with ICH

	Favorable outcome (mRS 0-3)								
	**Model 4**			**Model 5**			**Model 6**		
	**OR**	**95% CI**	***p***	**OR**	**95% CI**	***p***	**OR**	**95% CI**	***P***
**Clinical variables**									
NIHSS score on admission	0.865	0.79-0.95	0.003*	0.834	0.74-0.94	0.002*	0.826	0.71-0.95	0.010*
Admission hematoma volume (ml)	0.978	0.95-1.01	0.146	0.968	0.94-1.00	0.050	0.963	0.93-1.00	0.059
Admission eGFR (per 10 mL/min/1.73 m²)	0.992	0.97-1.02	0.488	0.975	0.95-1.00	0.084	0.979	0.94-1.02	0.267
Glasgow Coma Scale on admission	0.967	0.72-1.29	0.820	0.879	0.64-1.20	0.421	0.946	0.64-1.40	0.781
Hypertension	0.273	0.08-0.93	0.039*	0.345	0.10-1.24	0.104	0.390	0.08-1.97	0.255
Diabetes mellitus	2.134	0.63-7.23	0.223	2.424	0.63-9.32	0.198	17.541	2.33-131.83	0.005*
Atrial fibrillation	0.133	0.03-0.57	0.006*	0.258	0.05-1.28	0.098	0.164	0.02-1.42	0.101
Coronary artery disease	1.643	0.30-8.99	0.567	1.198	0.21-6.88	0.840	1.683	0.19-14.57	0.636
History of stroke	0.987	0.31-3.19	0.982	1.964	0.50-7.68	0.332	0.789	0.16-3.95	0.773
Chronic kidney disease	0.194	0.04-0.90	0.037*	0.144	0.03-0.74	0.020*	0.118	0.02-0.78	0.026*
Smoking	0.607	0.21-1.80	0.367	0.534	0.16-1.74	0.298	0.231	0.05-1.02	0.052
Alcohol abuse	1.596	0.33-7.81	0.564	1.328	0.23-7.76	0.753	2.347	0.26-21.14	0.447
Systolic blood pressure on admission (mmHg)	1.006	0.98-1.03	0.609	1.015	0.99-1.04	0.287	1.035	1.00-1.07	0.050
Diastolic blood pressure on admission (mmHg)	1.009	0.97-1.05	0.642	0.982	0.94-1.02	0.392	0.970	0.93-1.02	0.222
Age (years)	--			0.957	0.92-1.00	0.027*	0.947	0.90-1.00	0.054
Premorbid mRS	--			0.551	0.34-0.89	0.014*	0.553	0.31-0.99	0.050
**Admission laboratory variables (Model F only)**									
Glucose (mg/dL)	--			--			0.977	0.96-0.99	0.001*
Urea (mg/dL)	--			--			0.980	0.93-1.03	0.437
Potassium (mmol/L)	--			--			3.088	0.94-10.17	0.064
Sodium (mmol/L)	--			--			1.112	0.94-1.31	0.206
Leukocyte count (x10^3/uL)	--			--			1.005	0.82-1.24	0.965
Platelet count (x10^3/uL)	--			--			0.998	0.99-1.01	0.668
Hemoglobin (g/dL)	--			--			1.161	0.39-3.42	0.786
Hematocrit (%)	--			--			1.092	0.72-1.66	0.680
C-reactive protein (mg/L)	--			--			1.002	0.98-1.03	0.868

OR - odds ratio; CI - confidence interval; eGFR- estimated glomerular filtration rate; NIHSS - National Institutes of Health Stroke Scale; mRS – modified Rankin Scale; *p < 0.05; -- not included in the model.
